# Comparative Analysis of Fecal Microbiota in Infants with and without Eczema

**DOI:** 10.1371/journal.pone.0009964

**Published:** 2010-04-01

**Authors:** Pei-Ying Hong, Bee Wah Lee, Marion Aw, Lynette Pei Chi Shek, Gaik Chin Yap, Kaw Yan Chua, Wen-Tso Liu

**Affiliations:** 1 Division of Environmental Science and Engineering, National University of Singapore, Singapore, Singapore; 2 Department of Paediatrics, Yong Loo Lin School of Medicine, National University of Singapore, Singapore, Singapore; 3 Department of Civil and Environmental Engineering, University of Illinois at Urbana-Champaign, Urbana, Illinois, United States of America; University of Wisconsin-Milwaukee, United States of America

## Abstract

Eczema is a chronic form of childhood disorder that is gaining in prevalence in affluent societies. Previous studies hypothesized that the development of eczema is correlated with changes in microbial profile and composition of early life endemic microbiota, but contradictory conclusions were obtained, possibly due to the lack of minimization of apparent non-health related confounders (e.g., age, antibiotic consumption, diet and mode of delivery). In this study, we recruited seven caesarean-delivered and total formula-fed infants, and comparatively examined the early-life endemic microbiota in these infants with and without eczema. Using 16S pyrosequencing, infants' fecal microbiota were observed to comprise Proteobacteria, Firmicutes, Actinobacteria and Bacteroidetes as the four main phyla, and the presence and absence of specific populations within these four phyla are primarily mediated by ageing. Quantitative analysis of bacterial targets on a larger sample size (n = 36 at 1, 3, and 12 months of age) revealed that the abundances of *Bifidobacterium* and Enterobacteriaceae were different among caesarean-delivered infants with and without eczema, and the bacterial targets may be potential biomarkers that can correlate to the health status of these infants. Our overall findings suggest that the minimization of possible confounders is essential prior to comparative evaluation and correlation of fecal microbiota to health status, and that stool samples collected from caesarean-delivered infants at less than 1 year of age may represent a good cohort to study for potential biomarkers that can distinguish infants with eczema from those without. These findings would greatly facilitate future efforts in understanding the possible pathogenesis behind certain bacterial targets, and may lead to a timely intervention that reduces the occurrence of early life eczema and possibly allergic disorders in later life.

## Introduction

Eczema is a chronic form of childhood disorder that is characterized by remitting and relapsing cutaneous symptoms. These symptoms include itching and dryness, flaking, blistering, oozing, and bleeding. Together with other allergic disorders such as asthma and allergic rhinitis, the prevalence of eczema has been increasing globally, particularly in populations with a western lifestyle [1]. Although allergic disorders like eczema are usually not life-threatening, they impose considerable health and emotional burden on the child [2]. Eczema has a multifactorial etiology, and interaction of genetic factors with the environment has been shown to play an important role in the pathogenesis of allergic disorders [3]. Recent studies have also shown that early life endemic microbiota may play an important role in the subsequent development of allergic disorders [4], [5], [6]. For example, past observations show that in germ-free animal models, the correction of allergy response by the introduction of conventional gut microbiota was only achieved in neonates but not in older mice [7].

It is hypothesized that changes in the early life endemic microbiota may correlate to the health status of the infants. To understand the role of early life endemic microbiota in modulating health, their composition in healthy infant gut/feces was first analyzed through the use of 16S rRNA gene amplicons. Results suggest that fecal microbiota of infants are made up primarily of Actinobacteria, Proteobacteria, Firmicutes and Bacteroidetes [8], [9], [10]. These microbial groups not only provide for the host's metabolic needs but also promote the development of immune system that may in turn influence health status of the young hosts [11]. As we gain a better understanding of the endemic microbiota in healthy infants, it would be pertinent to question the differences in the endemic microbiota of infants with and without eczema (i.e., allergic disorder).

Previous findings have reported that infants with eczema appeared to have lower microbial diversity in their stools when compared to healthy controls, suggesting that alteration of the entire gut/fecal microbiota and the lack of exposure to certain bacterial targets may be contributing factors that amounted to their diseased state [12]. Nevertheless, contradictory results which showed no significant difference in total bacterial profiles of healthy and diseased infants have also been reported [13]. Comparative semi-quantitative analyses were used to identify potential eczema-related bacterial markers but no consensus was reached [13], [14], [15], [16]. Some studies reported that infants with allergic disorders tend to have lower abundance of bifidobacterial populations and higher proportions of *Escherichia coli* compared to the healthy controls [14], [15], while other studies showed no distinct differences in the bifidobacterial populations among infants with and without allergic disorders [13], [16].

One of the main objectives in the Human Microbiome Project is to determine whether changes in the human microbiome can be correlated with changes in the health status [4]. A common approach to address this is to perform a comparative evaluation on the fecal microbiota of healthy and diseased hosts. However, the infant fecal microbiota is highly dynamic and subjected to numerous non-health related confounding factors such as age of infant, mode of delivery, dietary regime and consumption of antibiotics [17]. Thus, minimizing the apparent non-health related confounders would be effective prior to evaluating the correlation between changes in fecal microbiota and the health status of infants.

In addition, past studies only monitored microbial succession in infants with conventional molecular methods like denaturing gel gradient electrophoresis (DGGE) and SSU rRNA gene-based microarray [8], [9], [10] that have their own technical limitations. DGGE has a detection limit of >1% of the total amplified genes, and subsequent identification by means of gene sequencing would only qualitatively denote the presence of predominant groups [18]. Similarly, SSU rRNA gene-based microarray only provides semi-quantitative detection of bacterial targets, and would not be able to detect unknown bacterial targets that are not included in the microarray [19]. Other advanced molecular methods such as 454 pyrosequencing would therefore serve as a better tool to examine microbial succession.

To systematically address the differences in the microbial community of infants with and without eczema, this study sampled stools from seven infants who were delivered via caesarean section and total-formula fed from birth (Table 1, Text S1). Three were clinically diagnosed with eczema and the severity assessed by SCORAD [20]. Using a 16S rRNA-based pyrosequencing [21], we then analyzed and compared the differences in microbial succession of these two health groups over four time points (i.e., 1, 3, 12 and 24 months of age), among which the stools from the first two time points were not affected by antibiotic treatment. All except one infant received antibiotics at the latter two time points, but the effect of antibiotic treatment on stool microbiota were kept to a minimum by sampling for feces at least 1 month after antibiotic consumption [22]. Through the comparison on the extent of differences in the microbial profile, we aimed to identify bacterial targets that were numerically abundant in either of the health groups, and examined their abundances in a larger infant cohort (n = 36 at 1, 3, and 12 months after birth, respectively) with a rapid and quantitative molecular tool [23]. These experiments would allow us to verify if identified potential biomarkers were representative of health status. The findings would provide a better understanding to whether differences in fecal microbiota correlate with the occurrence of eczema, and facilitate future efforts in understanding the possible pathogenesis behind eczema-related bacterial targets.

**Table 1:** Clinical data of the seven caesarean-delivered, total-formula-fed infant subjects.

Subject*	Dietary regime (From birth till 2 years)	Onset of eczema	SCORAD (month of diagnosis)	Skin Prick Test at 1 yr and 2 yr	Total IgE at 1 yr (kU/L)	Use of antibiotics (duration before sampling)
C-1	Total-formula	N.A.	N.A.	Negative	3.43	Cotrimoxazole (9 months before 12 months sampling)Cotrimoxazole (6 months before 24 months sampling)
C-2	Total-formula	N.A.	N.A.	Negative	33.40	Erythromycin and amoxicillin (6 months before 12 months sampling)
C-3	Total-formula	N.A.	N.A.	Negative	8.03	Azithromycin (3 months before 24 months sampling)
C-4	Total-formula	N.A.	N.A.	Negative	136.00	Nil
E-1	Total-formula	3 months	7.4 (3 months)	Positive for inhalant allergens (*Dermatophagoides pteronyssinus*)	323.00	Amoxicillin-clavulanic acid (1 month before 12 months sampling, 7 months before 24 months sampling)
E-2	Total-formula	6 months	10.9 (6 months)	Negative	5.66	Amoxicillin-clavulanic acid (7 months before 24 months sampling)Erythromycin (10 months before 24 months sampling)
E-3	Total-formula	1 month	21.2 (1 month)11.6 (6 months)	Negative	9.60	Amoxicillin-clavulanic acid (12 months before 24 months sampling)

*Stool samples were obtained from infants with and without eczema, and analyzed for their microbial community using 16S pyrosequencing.

## Results

### Microbial succession evaluated at phyla level

**Figure 1:**
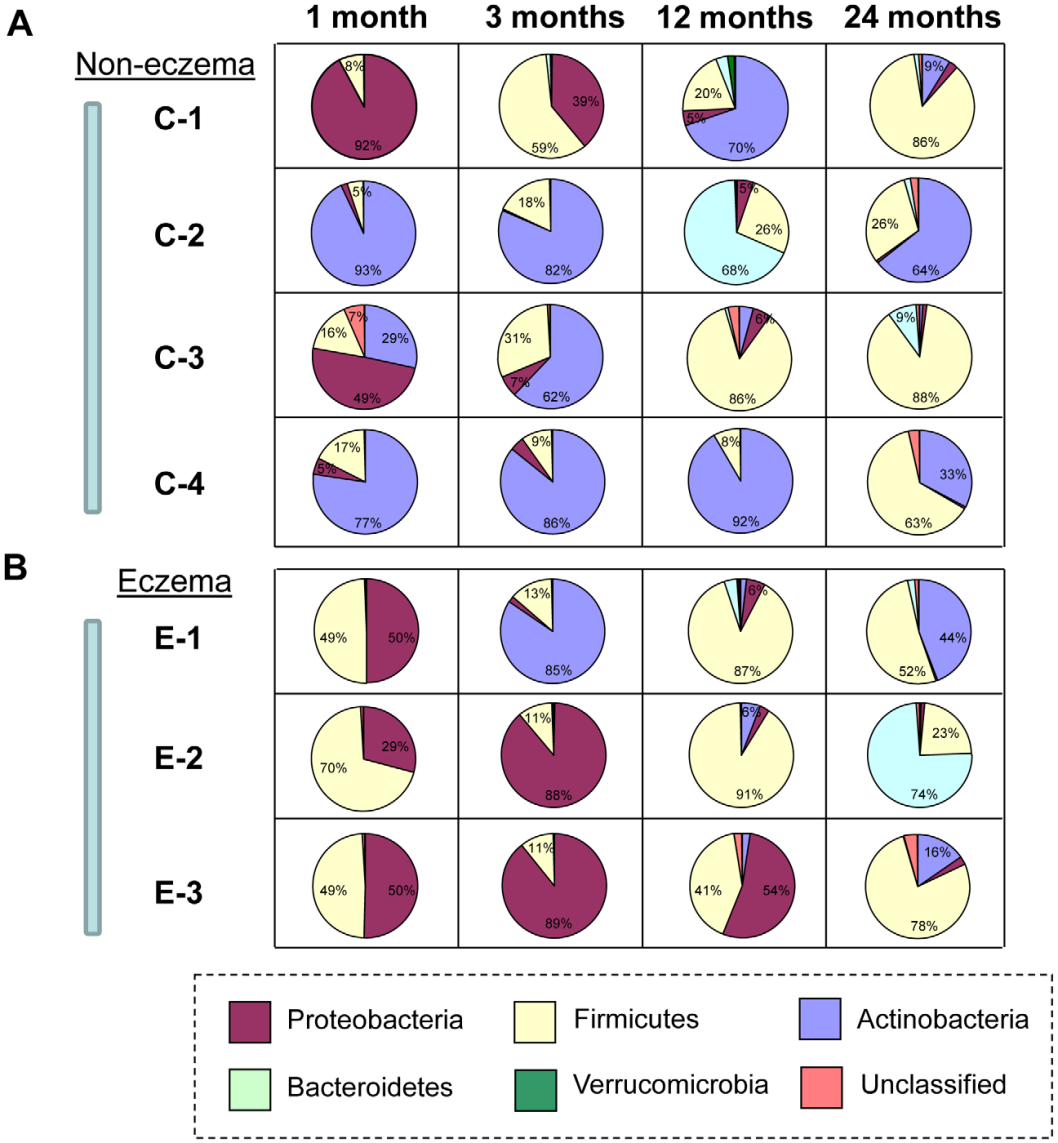
Microbial succession evaluated at phyla level with 454 pyrosequencing. (A) C-1, C-2, C-3 and C-4 are infants without eczema. (B) E-1, E-2 and E-3 are infants with eczema. The stools were comprised mainly of phyla Proteobacteria, Actinobacteria, Firmicutes, Bacteroidetes in varying abundances within each individual and across different temporal intervals. Phyla with abundances more than 5% are marked accordingly.

Microbial diversity and its succession within the seven infants was examined based on an average 4,396 16S rRNA amplicon sequences (also termed as 16S pyrotags) per treatment. At 1 month of age, Proteobacteria was detected in feces of all individuals and accounted for 1.8 to 92.2% of 16S pyrotags. In particular, Proteobacteria constituted one of the two predominant phyla that was present in the fecal microbiota of non-eczema infant C-1 (Figure 1A) and all eczema infants at 1 month of age (Figure 1B). With increasing age, the proportion of Proteobacteria decreased, with the exception of two eczema infants E-2 and E-3 at 3 months of age (Figure 1B). By the age of 24 months, Proteobacteria made up less than 1.4±1.0% of 16S pyrotags. Firmicutes was another predominant phylum that was present in the fecal microbiota of all infants with eczema. It was consistently detected at all time points but its succession varied differently among individuals. For example, Firmicutes made up 48.9 to 70.2% of 16S pyrotags from the eczema infants at 1 month of age. Its abundance in the eczema infants rapidly decreased to 11.4±1.6% at 3 months, and increased again to 73.1+27.5% at 12 months of age. At 24 months, abundances of Firmicutes decreased in E-1 and E-2 but increased in E-3 (Figure 1). Actinobacteria consistently remained as the predominant phylum in three of the non-eczema infants (i.e., C-2, C-3 and C-4) and accounted for 28.5 to 93.1% of 16S pyrotags at 1 and 3 months of age. However, it was clearly absent in feces sampled from eczema infants at 1 and 3 months of age, with the exception of E-1 at 3 months. At 12 months onwards, the proportion of Actinobacteria decreased with time for all infants and was gradually succeeded by Bacteroidetes. The abundance of Bacteroidetes as observed in C-2, where the abundance decreased drastically from 68% of 16S pyrotags at 12 months to 2.1% of 16S pyrotags at 24 months of age, was not stable. Verrucomicrobia is a rare phylum that was only observed in selected individuals at <2% of 16S pyrotags and at a particular time-point. Similarly, unclassified bacteria only accounted up to 4.3% of 16S pyrotags.

### Microbial succession evaluated at genus level

**Figure 2:**
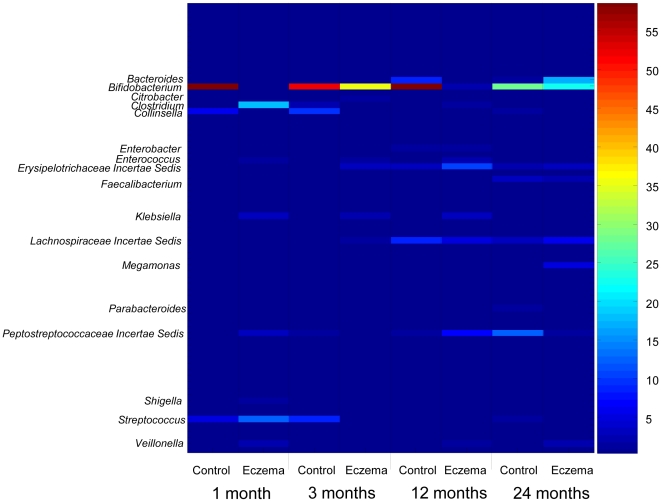
Color-coded heat plot for the microbial succession evaluated at genera level with 454 pyrosequencing. The color intensity of the heat plot denotes the average percentage abundance associated with the bacterial target. Control group denotes the four non-eczema healthy infants, Eczema group denotes the three eczema infants.

RDP Classifier was used for taxonomical assignments of the aligned 16S pyrotags at 95% confidence level. Based on the heat plot that color-coded the range of abundances (Figure 2), the phylum Proteobacteria was made up of mainly genera *Citrobacter*, *Klebsiella, Shigella*, and *Enterobacter*, with the first three genera detected only in eczema infants but not in non-eczema infants. This result suggested that although Proteobacteria was detected consistently in all infants, its composition differed among eczema and non-eczema infants. The phylum *Firmicutes* was made up of various genera like *Clostridium*, *Enterococcus*, *Erysipelotrichaceae Incertae Sedis*, *Faecalibacterium*, *Lachnospiraceae Incertae Sedis*, *Megamonas*, *Peptostreptococcaceae Incertae Sedis*, *Streptococcus* and *Veillonella*. At different stages of infancy, different genera within Firmicutes predominated over others. Phylum Actinobacteria was made up of only *Bifidobacterium* and *Collinsella*, with *Bifidobacterium* dominating in the early infancy. Phylum Bacteroidetes consisting of *Bacteroides* and *Parabacteroides* was detected from 12 months onwards, suggesting a maturation of the gut/fecal microbiota.

### Age-related changes in microbial richness and diversity

**Figure 3:**
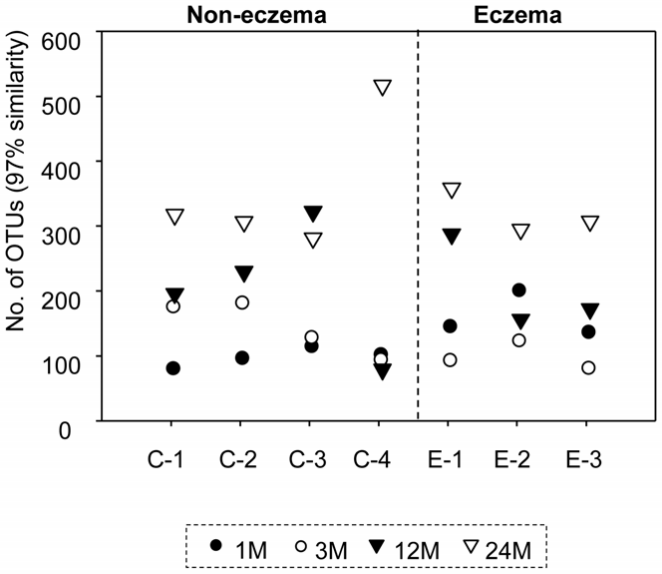
Individual changes in OTUs in relation to time. The number of operational taxonomic units (OTUs) defined at 97% similarity was based on 6000-pyrotag reads. The number of OTUs increased with age.

**Figure 4:**
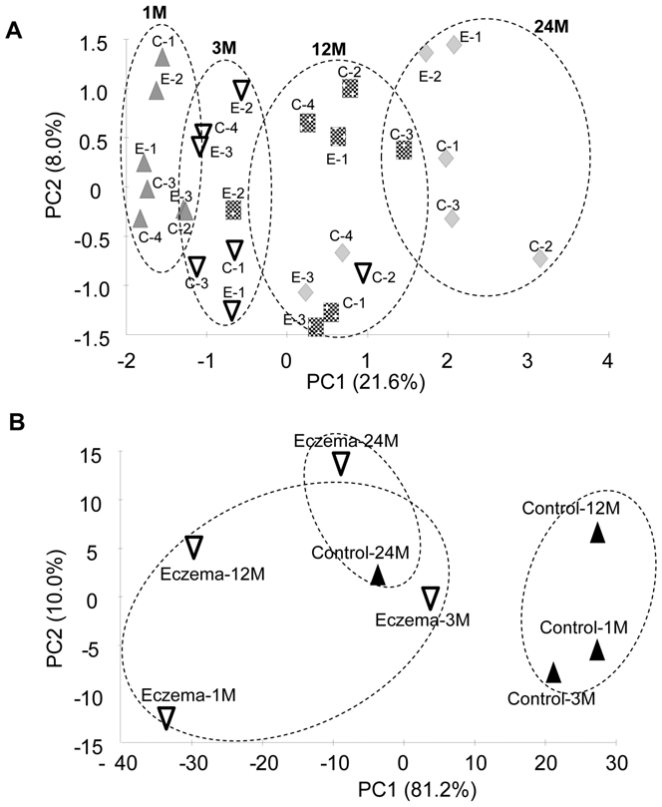
Principal component analysis (PCA). (A) PCA was performed based on the presence and absence of OTUs identified in each individual at different time-points: upward triangle represents samples at 1 month; downward triangle represents samples at 3 months; square represents samples at 12 months; kite represents samples at 24 months. PCA indicated that presence and absence of OTUs are time-mediated. (B) PCA was performed based on abundance of classified OTUs within the eczema and non-eczema control health groups at different time-points. PCA indicated that abundances of specific bacterial targets correlated to the eczema status of infants during early stages of infancy.

For individual treatments, rarefraction curves that defined the number of operational taxonomic units (OTUs at 97% gene similarity) with respect to the number of sequenced 16S pyrotags were plotted (Figure S1). Regression analysis of the rarefraction curve allows a subsequent computation of the number of OTUs per treatment that is identified based on 6,000 16S pyrotags (Table S1). As illustrated in Figure 3, the number of OTUs increased significantly from 130±39 at 1 month of age to 344±81 at 24 months of age. The number of OTUs in eczema infants at 3 months of age was 1.6-fold lower than at 1 month of age, but increased to a similar level as non-eczema infants after 12 months (P-value = 0.716) (Figure 4). Among the non-eczema infants, C-4 exhibited a different trend in the change of OTUs with age. For instance, the number of OTUs was consistently low in the first 12 months, and increased by nearly five-fold to 520 OTUs at 24 months of age. We noted that the infant had high serum total IgE of 136 kU/L (Table 1) at 12 months of age, and speculated that the raised IgE may be associated with the lower number of OTUs before 12 months of age. We matched all OTUs to its phylogenetic affiliation, and used this information to generate a principal component analysis (PCA) that is based on the presence and absence of individual OTUs in individual samples. A total of 27 dimensions were used to account for the total variance. The first two dimensions PC1 and PC2, which accounted for 29.6% of the total variance did not show an apparent correlation to the eczema status of the infants (Figure 4A). The first dimension (21.6%) was correlated to age, suggesting that the ageing process was a relatively domineering factor that mediated changes in microbial richness and diversity among infants.

### Comparison of bacterial lineages and their abundances in infants

We further compared bacterial lineages and their abundances in infants at each time point to evaluate if abundances of classified OTUs correlated with eczema in infants. The PCA results (Figure 4B) indicate that the first two dimensions accounted for a total of 91.2% of variance, and a clustering effect based on health status could be observed along the PC1 dimension. In particular, non-eczema groups were clustered apart from eczema groups at 1, 3 and 12 months of age. This suggested that during early stages of infancy (i.e., below 12 months of age), abundances of classified OTUs may differentiate infants with eczema from the healthy matched controls. To further identify bacterial lineages that were significantly abundant in eczema infants or healthy controls, the 16S pyrotag libraries were compared in RDP LibCompare. *Bifidobacterium* was present at significantly higher abundance in non-eczema infants compared to those with eczema at all time points (P-value <0.001), while *Enterococcus*, *Klebsiella* and *Shigella* were present at significantly higher abundances in eczema infants during early stages of infancy (P-value <0.001).

### Abundances of potential biomarkers in vaginal-delivered infants

**Figure 5:**
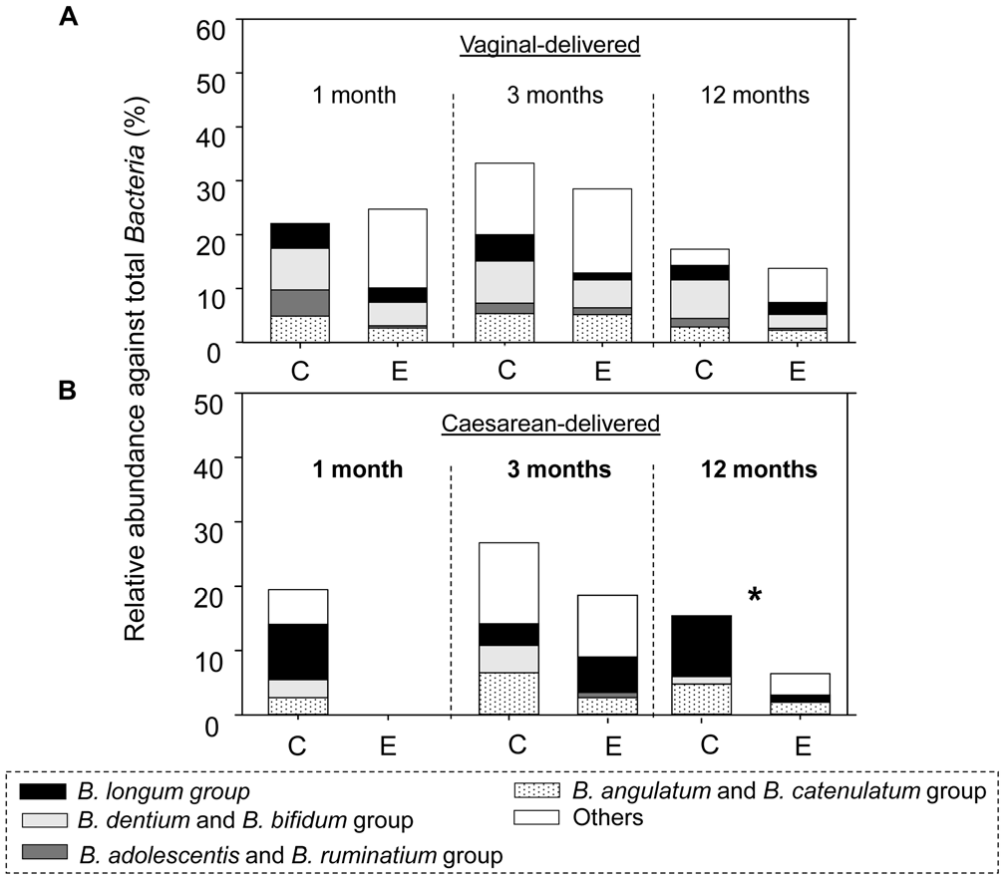
Abundances of individual species within the bifidobacterial population as quantified by HOPE. (A) Abundances found in vaginal-delivered infants (n = 27), (B) Abundances found in caesarean-delivered infants (n = 14). Abbreviation C stands for non-eczema control infants, E stands for infants with eczema. * indicates significant differences in the *B. longum* group found in non-eczema and eczema infants at 12 months of age.

A total of 27 vaginal-delivered babies (14 non-eczema controls and 13 eczema infants) were sampled for their feces at 1, 3 and 12 months of age. To evaluate if *Bifidobacterium*, *Enterococcus* and Enterobacteriaceae (including *Klebsiella* and *Shigella*) could serve as potential biomarkers that are present in different abundances among eczema and non-eczema infants, their relative abundances in the collected feces were quantified using a rapid molecular tool (HOPE, hierarchical oligonucleotide primer extension) developed for microbial source tracking and community analysis [24], [25]. The results showed that at the time points sampled, the relative abundances of *Bifidobacterium* spp. in feces of non-eczema infants ranged from 17.3 to 33.3%, while the abundances in eczema infants ranged from 13.7 to 28.5% (Table 2). Although abundances of genus *Bifidobacterium* was 1.2 and 1.3-fold higher in non-eczema infants than eczema infants at 3 and 12 months of age, respectively, the differences were not significant (P-value = 0.35 and 0.17, respectively) (Table 2). The abundances of individual *Bifidobacterium* spp. were also not significantly different among vaginal-delivered eczema and non-eczema infants (Figure 5A). Similarly, the relative abundances of *Enterococcus* and Enterobacteriaceae against the total *Bacteria* were not significantly different among vaginal-delivered infants of both health groups (Table 2).

**Table 2:** Comparison of potential biomarkers present in feces of infants as quantified by HOPE.

Vaginal-delivered infants (n = 27)		*Bifidobacterium*		*Enterococcus*		Enterobacteriaceae	
		Non-eczema	Eczema	Non-eczema	Eczema	Non-eczema	Eczema
1 month	Frequency of occurrence (%) ^	57.1	84.6	64.3	53.8	35.7	38.5
	Average abundances *	21.5±24.0	24.7±17.0	3.2±4.3	2.5±3.7	3.6±5.5	2.0±3.2
	Ratio of abundances #	0.87		1.28		1.80	
	P-value	0.39		0.32		0.36	
3 months	Frequency of occurrence (%)	92.9	83.3	71.4	50.0	35.7	16.7
	Average abundances	33.3±18.5	28.5±20.7	2.6±2.9	1.2±1.8	1.0±1.5	0.4±0.9
	Ratio of abundances	1.17		2.17		2.50	
	P-value	0.35		0.09		0.12	
12 months	Frequency of occurrence (%)	84.6	90.9	53.8	63.6	7.7	0.0
	Average abundances	17.3±11.5	13.7±8.0	0.8±1.1	0.9±0.8	0.2±0.7	N.A.
	Ratio of abundances	1.26		0.89		N.A.	
	P-value	0.17		0.31		N.A.	

Caesarean-delivered infants (n = 14)		*Bifidobacterium*		*Enterococcus*		Enterobacteriaceae	
		Non-eczema	Eczema	Non-eczema	Eczema	Non-eczema	Eczema
1 month	Frequency of occurrence (%)	62.5	0.0	12.5	75.0	12.5	75.0
	Average abundances	19.2±18.1	N.A.	2.4±2.5	4.0±4.8	0.3±0.8	4.0±4.4
	Ratio of abundances	N.A.		0.60		0.08	
	P-value	N.A.		0.27		0.02 ×	
3 months	Frequency of occurrence (%)	75.0	50.0	25.0	100.0	25.0	100.0
	Average abundances	26.6±20.2	18.4±22.1	2.9±4.0	4.5±3.9	1.0±2.1	4.7±3.2
	Ratio of abundances	1.45		0.64		0.21	
	P-value	0.28		0.32		0.01	
12 months	Frequency of occurrence (%)	62.5	66.7	62.5	66.7	0.0	16.7
	Average abundances	14.0±14.6	6.3±7.6	3.3±6.1	2.2±3.9	N.A.	2.7±6.5
	Ratio of abundances	2.22		1.50		N.A.	
	P-value	0.23		0.50		N.A.	

^ Frequency of occurrence denotes the percentage number of infants who were detected with the bacterial target in their stools.

* Average abundance is obtained by summing up abundances of bacterial target in all individuals of that particular health group, and the total sum divided by the number of individuals.

# Ratio of abundances is expressed by dividing average abundance of bacterial target in non-eczema group against the eczema-group.

× Underlined P-values indicate significant difference at 95% confidence level.

### Abundances of potential biomarkers in caesarean-delivered babies

A total of 14 caesarean-delivered babies (8 non-eczema controls and 6 eczema infants) were sampled for their feces at 1, 3 and 12 months of age. Within these samples, the relative abundances of *Bifidobacterium*, *Enterococcus* and Enterobacteriaceae against the total *Bacteria* were quantified. Unlike the vaginal-delivered infants, the findings suggested that all of the caesarean-delivered eczema infants had a delayed occurrence of bifidobacterial population that was only detected at 3 months of age (Table 2). Although relative abundances of *Bifidobacterium* against the total *Bacteria* were not significantly different among the caesarean-delivered eczema and non-eczema infants (P-value >0.23), the abundances in the eczema infants were at least 1.5-fold lower than in the non-eczema infants (Table 2). *Bifidobacterium* spp. present in feces of caesarean-delivered and eczema infants were less diverse than the non-eczema group at 1 month of age. For example, *B. angulatum*, *B. adolescentis*, *B. dentium*, the *B. catenulatum* group, the *B. bifidum* group and the *B. longum* group were only detected in non-eczema infants at 1 month of age, and accounted for 2.5 to 8.6% of the total *Bacteria* (Figure 5B). At 3 months of age, specific *Bifidobacterium* spp. like *B. dentium* and the *B. bifidum* group were only detected at an average abundance of 4.2% in non-eczema infants but not in eczema infants (Figure 5B). At 12 months of age, the relative abundances of the *B. longum* group was significantly higher in non-eczema infants than eczema infants (P-value = 0.05) (Figure 5B). In contrast, at 1 and 3 months of age, the relative abundances of *Enterococcus* against the total *Bacteria* were lower in non-eczema infants than in eczema infants. The relative abundance of Enterobacteriaceae was significantly lower in the non-eczema infants than in eczema infants (P-value = 0.02 and 0.01, respectively) (Table 2).

## Discussion

This study aims to utilize 16S pyrosequencing to provide an in-depth analysis of microbial succession in infants. The study also aims to identify potential bacterial targets that can distinguish infants with and without eczema. The results reveal that Proteobacteria, Firmicutes, Actinobacteria and Bacteroidetes constitute the four main phyla in stools sampled from total-formula-fed and caesarean-delivered infants. Proteobacteria is generally regarded as the primary colonizer in infants during delivery, and may originate from the skin microbiota of mothers and hospital personnel [9], [26]. The origins of Firmicutes and Actinobacteria were perceived to be mainly transmitted to infants from mothers through their vaginal microbiota and breast milk, respectively [27], [28]. Our findings however showed that caesarean-delivered and total-formula-fed infants also had a microbial composition that is similar to the vaginal-delivered and breast-fed infants by 1 month of age, even though they are not exposed to microorganisms via the conventional vagina and breast-milk routes. This observation suggests that infants also receive microorganisms from their mothers during gestation in addition to mothers' vaginal microbiota and breast milk as the main sources of microbial inoculums. Together with other findings which reported that microorganisms like *Bifidobacterium* spp. and *Lactobacillus* spp. were found to be present in low abundances in the placenta and meconium of neonates [29], [30], [31], we think that gastrointestinal tracts of newborn infants are not as sterile as previously thought.

It is reported that after the pre-colonization period, different microorganisms subsequently establish and predominate over a “conserved and conventional program” that is in part mediated by numerous factors such as age, individual genetics, diet and environmental settings [9], [17]. In addition, there may be other confounders that can potentially affect microbial succession. Though delivery mode and diet among all sampled infants were kept similar, and that the effect of antibiotics treatment were kept minimal in this study, we could still identify as many as 27 dimensions to account for the total variance that correlated to the presence and absence of OTUs in infants' fecal microbiota. Among them, the age of infant was a significant factor that accounted for 21.6% of PCA variance (Figure 4A), while other confounders such as host genetics, consumption of antibiotics, slight dietary differences during weaning and health status of the infants may account for small portions of the total PCA variance.

Our findings suggest that the presence and absence of OTUs did not correlate significantly to the health status of the infants. The number of unique OTUs identified based on 6000-pyrotags was not significantly different in the non-eczema and eczema infants at all time points (Figure 3). Although there was antibiotics consumption during the late stage of infancy, there was no observable adverse effect on the OTU increment with time (Figure 3). This observation seems to suggest the resilience of early life endemic microbiota in some individuals after short term antibiotic administration [22], [32]. PCA analysis performed using presence and absence of OTUs suggested that the first dimension of 21.6% variance could not be related to eczema, but was instead age-mediated (Figure 4A). These observations are in agreement with those reported by Penders and coworkers [13], who also did not observe significant differences in the number of DGGE bands among non-eczema and eczema infants at 1 month of age. However, Wang and coworkers [12] reported a significantly lower microbial diversity in the feces of 1 week old eczema infants than in healthy subjects by comparing the number of peaks and bands found on the T-RFLP and TGGE profiles. These contradictory observations suggest that microbial richness may not be a consistent reliable parameter to distinguish eczema infants from non-eczema ones, even after the attempt to minimize non-health related confounders.

In contrast, PCA analyses performed based on the abundances of individual OTUs convincingly suggested a strong correlation between abundances of specific bacterial genera and health status of the infants during early stages of infancy. *Bifidobacterium* was observed to be present at significantly higher abundances in the caesarean-delivered non-eczema infants than eczema infants, and vice versa for *Enterococcus*, *Klebsiella* and *Shigella*. This observation was closely aligned to previous studies [15], [33], [34], [35] that reported a higher abundance of *Bifidobacterium* spp. in non-eczema infants compared to those with eczema, as well as a higher occurrence frequency of enterococci and Enterobacteriaceae in eczema infants.

To verify if *Bifidobacterium*, *Enterococcus* and Enterobacteriaceae are potential biomarkers that can be used to distinguish infants with eczema from those without, their abundances in a larger sample size were quantified using HOPE. The results suggested that the abundances of these identified bacterial targets were not significantly different in the vaginal-delivered non-eczema and eczema infants. However in caesarean-delivered infants, Enterobacteriaceae was found at significantly higher abundances in the eczema infants compared to non-eczema ones (P-value <0.02) (Table 2). Although its abundances were not significantly higher among the caesarean-delivered non-eczema than eczema infants, *Bifidobacterium* was only detected in the non-eczema infants at 1 month of age, and was present in more than 1.4-fold higher abundance compared to the eczema infants at other time points (Table 2). The observed difference in abundance of *Bifidobacterium* and Enterobacteriaceae in caesarean-delivered infants but not in vaginal-delivered ones, can possibly explain why probiotics supplementation would have an effect on the former cohort and not the latter [36]. As this study only included a limited sample size for HOPE analysis, future studies with larger sample size will have to be conducted to provide more conclusive evidence to support this observation.

Nevertheless, a closer examination of the bifidobacterial population within the caesarean-delivered infants further showed differences in relation to the host health status. Among those caesarean-delivered infants, *B. adolescentis* group was only detected in the eczema infants at 3 months of age, but not in the non-eczema ones (Figure 5B). It is reported that *B. adolescentis* has lower adhesive ability compared to other bifidobacterial population, and this can potentially result in an aberrant endemic microbiota and its associated immune response in caesarean-delivered eczema infants [37]. The *B. longum* group in contrast was only detected in fecal microbiota of caesarean-delivered non-eczema infants at 1 month of age. Its abundance was also up to 3.8-fold higher in the stools of non-eczema infants than eczema ones throughout the sampled period (Figure 5B). These findings suggested that different species within *Bifidobacterium* may modulate health status in a different manner, although overall abundance of this genus is higher in non-eczema infants than in eczema infants.

This study assumes that a better understanding of the early life microbiota in relation to the host health status can lead to a timely intervention that can possibly reduce the occurrence of diseases in later life. Based on this, eczema and non-eczema infants with known clinical characteristics were recruited, and their fecal microbiota were comparatively evaluated with different molecular tools. We observed that the presence and absence of OTUs in the fecal microbiota was closely associated with ageing and not to the infant health status. We further observed that abundances of specific bacterial lineages (e.g., *Bifidobacterium*, *Enterococcus* and Enterobacteriaceae) were closely associated with the occurrence of eczema in caesarean-delivered infants after minimization of apparent non-health related confounders. In particular, stools collected from caesarean-delivered infants before 12 months of age may represent a good model to study for potential biomarkers that can distinguish non-eczema and eczema infants. Future studies can utilize a similar experimental approach to examine potential biomarkers in a larger sample size of vaginally-delivered infants. This would further verify if specific biomarkers are good diagnostic markers that can identify infants at risk of developing eczema. In summary, the findings would greatly facilitate future efforts in understanding the possible pathogenesis behind eczema-related bacterial targets, and may lead to a timely intervention that reduces the occurrence of early life eczema and possibly allergic disorders in later life.

## Materials and Methods

### Ethics statement

Written informed consent was obtained from all families. The study was approved by the National University Hospital's ethics review committee (Ref Code: B/00/322).

### Infant subjects and clinical diagnosis

All subjects recruited for this study were of gestational age above 35 weeks and have a birth weight above 2 kg with no major congenital malformations or illness at birth. With reference to Text S1, they belonged to placebo group of the clinical trial (ClinicalTrials.gov Identifier NCT00318695) and were not supplemented with probiotics or prebiotics [38]. Eczema was diagnosed by a doctor, and subjects did not have wheezing, asthma and allergic rhinitis. SCORAD values were assigned for all eczema infants [20]. Skin prick tests were performed at 12 and 24 months of age to assess for sensitization to common food and inhalant allergens. Serum total immunoglobin-E (IgE) was tested at first year after birth. Information on their respective diet and antibiotics treatment during the first 2 years of infancy were also obtained (Table 1). The stool samples examined in this study were a sub-cohort of the clinical trial, and were carefully chosen by minimizing potential non-health related confounders (Text S1). Seven infants who were caesarean-delivered and total formula-fed were selected to examine for their microbial diversity based on 454 pyrosequencing. Among them, three (E-1, E-2 and E-3) had onset of eczema in infancy and four (C-1, C-2, C-3 and C-4) were healthy controls. When assessed for sensitization to common food and inhalant allergens at 12 and 24 months of age, only E-1 showed sensitization to dust mite allergen (*Dermatophagoides pteronyssinus*) at 24 months. To further evaluate for differences in the abundances of *Bifidobacterium* spp., *Enterococcus* spp. and Enterobacteriaceae, a larger sampling size comprising 19 eczema infants were also included along with 22 non-eczema controls. All non-eczema controls were matched for their mode of delivery and diet regime throughout the sampling duration. The stools were collected at 1, 3, 12- and 24 months of age based on sampling procedure as described previously [39].

### DNA extraction and PCR

Bacterial DNA from stools was extracted as described previously [39]. Concentration of bacterial DNA was measured using DU800 spectrophotometer (Beckman Coulter, CA) and diluted to 50 ng/µl. Samples for 454 FLX pyrosequencing were amplified with bacterial-specific forward 47F (5′-Fusion A-Barcode-CA linker-GCYTAAYACATGCAAGT-3′) and reverse 534R (5′-Fusion B-TC linker-ATTACCGCGGCTGCTGGC-3′) primer pairs. A total of 28 bar-coded [40] forward primers were used to differentiate the individual samples. Reaction mixtures comprised of 100 ng of genomic DNA, 25 µl of Premix F (Epicentre Biotechnologies, WI), 200 nM (each) of forward and reverse primers, 0.5 U of Ex *Taq* DNA polymerase (Takara Bio Inc., Japan), and the volume added up to 50 µl with molecular-biology grade water. PCR with 30 cycles of thermal program (denaturation, 95°C for 30 s; annealing, 50°C for 45 s; and extension, 72°C for 60 s) was performed. Samples for HOPE were amplified based on protocol described previously with forward 11F [5′-GTT YGA TYC TGG CTC AG-3′] and reverse 1492R [5′- GGY TAC CTT GTT ACG ACT T-3′] primers [25]. All amplicons were gel-excised, concentrated and purified with Wizard DNA purification kit (Promega, WI). The concentrations were then measured by Qubit fluorometer (Invitrogen, CA) and DU800 (Beckman Coulter, CA).

### 454 pyrosequencing and pyrosequences alignment

454 pyrosequencing was carried out on 454 Life Science Genome Sequencer GS FLX (Roche, Switzerland). The services were provided by Roy J. Carver Biotechnology Center, University of Illinois at Urbana Champaign. A total of 123,097 pyrotags were obtained from 454 FLX pyrosequencing run, and each sequence has an average read length of 230 bp. The 16S pyrotags were sorted based on their respective barcodes to form a total of 28 pyrotag libraries. All 16S pyrotags were then removed of their barcodes, linker and fusion adaptor sequences. Two different alignment schemes were used. The first alignment is based on NAST which removes non-16S pyrotags in the returned alignment. As NAST server limits each submission to 500 pyrotags, an individual library was split into smaller subsets comprising 500 pyrotags, aligned with minimum length set at 200 bp, and with other settings kept at their default values [41]. After NAST alignment, the aligned subset libraries were merged back into a fasta format. The second alignment is based on RDP Infernal which allows secondary structure alignment [42]. To prevent loss of 16S pyrotags from the respective alignment schemes, we utilize a merge tool available at http://acai.igb.uiuc.edu/bio/ to merge aligned sequences from both NAST and RDP Infernal into one alignment file. The merge process further improves the alignment by replacing the long unaligned portions of RDP Infernal with the corresponding region from the NAST alignment. The aligned pyrotags were visually checked with Jalview: http://www.jalview.org/Web_Installers/install.htm, and manual adjustments were performed to improve the alignment whenever necessary.

### Taxonomical classification and statistical analysis

RDP Classifier was used for taxonomical assignments of the aligned 16S pyrotags at 95% confidence level [42]. Primer-E worksheets that detailed the presence and absence, as well as the percentage abundances of individual bacterial genera were collated, and subsequently performed with principal component analysis on Primer-E software (http://www.primer-e.com/).

### Rarefraction curves

Merged alignments were generated with their individual cluster files based on the RDP pyrosequencing pipeline [42]. The cluster files were in turn used to generate rarefraction curves that defined the number of operational taxonomic units (OTUs) defined at 97% similarity level with respect to the total number of pyrotags read (Figure S1). Regression analysis was also performed on Sigma Plot to fit the rarefraction curves into double rectangular hyperbola curve models (Table S1). Based on the curve models, the number of OTUs (97% gene similarity) identified based on 6000 pyrotags were noted for comparison in Figure 3.

### HOPE

Primers targeting at domain *Bacteria*, genus *Bifidobacterium* and its different clusters, *Enterococcus* spp., and most Enterobacteriaceae were arranged into two multiplexing HOPE reaction tubes (Table S2). HOPE reactions and capillary electrophoresis were carried out as described previously [23]. Fragment sizes and peak areas of the extended primers were recorded for subsequent calculation of the calibration factors (CF) and relative abundances of microbial targets. Calibration factors (CF) for a lower ranked primer with respect to a higher ranked primer can be obtained using the M13 amplicons of associated reference strains as template, and calculated as follow:
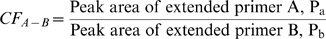
(i)


Where primer B is targeting at a higher hierarchical level compared to primer A.

The relative abundance of 16S rRNA gene amplicons targeted by the primer A with respect to those targeted by primer B can then be calculated as follow:
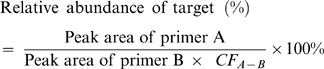
(ii)


### Statistical analysis for HOPE data

Mann-Whitney test was used to evaluate for significant differences in the medians of two groups of data at a confidence level of 90%. The non-parametric test was performed with Minitab.

## Supporting Information

Text S1 This file describes the selection protocol for infant subjects.
(0.04 MB DOC)

Table S1 Regression analyses of rarefraction curves.
(0.07 MB DOC)

Table S2 List of HOPE primers used in this study.
(0.05 MB DOC)

Figure S1 Rarefraction curves.
(1.31 MB PDF)
