# Supplementary material for: Comparative Analysis of Fecal Microbiota in Infants with and without Eczema
Source: PLoS One. 2010 Apr 1;5(4):e9964. doi: 10.1371/journal.pone.0009964 (PMC2848600; doi:10.1371/journal.pone.0009964)
Supplement: Text S1 — This file describes the selection protocol for infant subjects. (0.04 MB DOC) [file pone.0009964.s001.doc]

**Clinical trial NCT00318695**

- Recruited a total of 253 infants over 2 years (Soh et al., 2009)

**126 infants** not affected by probiotics and prebiotic treatment

- Stool samples suitable for use in this study

**127 infants** affected by probiotics and prebiotic treatment

- Stool samples not suitable for use in this study

**81** healthy infants

**45** infants diagnosed with allergic disorders like eczema, rhinitis and asthma

**28** infants with eczema

**19** infants with stools collected at time points 1, 3, 12 and 24 months

**9** infants with stools collected at incomplete time points, not suitable for use in this study

Total formula fed at all time points

**(n=3)**

Vaginal delivered

**(n=13)**

Caesarean section

**(n= 6)**

Total formula fed at all time points

**(n=1)**

Breast fed and formula fed

**(n=12)**

Breast fed and formula fed

**(n=3)**

Infants had no antibiotic consumption at 1 and 3 months of age. Short term (~5d) antibiotic administration occurred after ~12 months of age. Endemic microbiota is hypothesized to be resilient to short term antibiotic administration and would regain back its original microbial diversity after 1 month of antibiotic administration (De La Cochetiere et al., 2005; Dethlefsen et al., 2008)

Infants (i.e., E-1, E-2, E-3) were chosen for pyrosequencing along with their healthy matched controls (i.e., C-1, C-2, C-3, C-4)

**HOPE**

All 19 infants with eczema were included for HOPE, along with 22 healthy matched controls

(see legend ^)

^ Legend:

All 19 eczema infants displayed in the boxed up region were quantified for *Bifidobacterum* and Enterobacteriaceae using hierarchical oligonucleotide primer extension (HOPE) method. Appropriate healthy matched controls, with the same mode of delivery and dietary regime, were chosen accordingly. A total of 27 vaginal-delivered (i.e., 13 infants with eczema, 14 infants without eczema), and 14 caesarean-delivered infants (i.e., 6 infants with eczema, 8 infants without eczema) were included in HOPE analyses.

References cited in this supplementary text:

1. Soh et al. (2009) Probiotic supplementation in the first 6 months of life in at risk Asian infants – effects on eczema and atopic sensitization at the age of 1 year. Clin Exp Allergy 39: 571-578.
2. De La Cochetiere et al. (2005) Resilience of the dominant human fecal microbiota upon short-course antibiotic challenge. J Clin Microbiol 43: 5588-5592.
3. Dethlefsen et al. (2008) The pervasive effects of an antibiotic on the human gut microbiota, as revealed by deep 16S rRNA sequencing. PLoS Biol 6: e280.
