# Supplementary material for: Comparative Analysis of Fecal Microbiota in Infants with and without Eczema
Source: PLoS One. 2010 Apr 1;5(4):e9964. doi: 10.1371/journal.pone.0009964 (PMC2848600; doi:10.1371/journal.pone.0009964)
Supplement: Table S1 — Regression analyses of rarefraction curves. (0.07 MB DOC) [file pone.0009964.s002.doc]

**Table S1.** Regression analyses of rarefraction curves. Double hyperbola curve model was chosen to describe the trajectory of the rarefraction curves. The number of OTUs (97% similarity) was estimated based on 6000 pyrotags read.

| Double rectangular hyperbola curve is described by the following algorithm:  y = ax/(b+x) + cx/(d+x) + ex  where y = number of OTUs defined at 97% gene similarity  x = number of 16S pyrotags sequenced  a, b, c, d and e = numerical constants | | | | | | | |
| --- | --- | --- | --- | --- | --- | --- | --- |
| Sample | Time point (month) | a  (X10^1^) | b  (X10^1^) | c  (X10^1^) | d  (X10^2^) | e  (X10^-3^) | Value of y when x = 6000 |
| C-1 | 1 | 1.29 | 1.55 | 2.48 | 4.32 | 8.97 | 90 |
| C-1 | 3 | 4.02 | 5.75 | 8.84 | 15.0 | 11.5 | 179 |
| C-1 | 12 | 4.09 | 11.1 | 17.0 | 29.9 | 7.61 | 199 |
| C-1 | 24 | 5.49 | 9.62 | 21.0 | 20.3 | 18.4 | 321 |
| C-2 | 1 | 1.98 | 1.04 | 5.96 | 12.7 | 5.25 | 100 |
| C-2 | 3 | 2.73 | 5.46 | 17.4 | 21.3 | 5.00 | 185 |
| C-2 | 12 | 1.30 | 4.13 | 12.8 | 15.0 | 19.7 | 233 |
| C-2 | 24 | 5.37 | 11.9 | 23.9 | 27.5 | 15.5 | 310 |
| C-3 | 1 | 1.43 | 1.61 | 4.15 | 7.03 | 11.2 | 118 |
| C-3 | 3 | 2.99 | 10.8 | 12.3 | 32.8 | 3.91 | 132 |
| C-3 | 12 | 6.88 | 11.7 | 11.9 | 20.9 | 28.4 | 326 |
| C-3 | 24 | 6.68 | 8.54 | 19.1 | 27.8 | 14.7 | 285 |
| C-4 | 1 | 2.48 | 6.48 | 5.43 | 18.0 | 6.56 | 106 |
| C-4 | 3 | 9.90 | 3.61 | 6.60 | 18.5 | 6.26 | 98 |
| C-4 | 12 | 1.24 | 4.44 | 6.95 | 22.7 | 3.28 | 82 |
| C-4 | 24 | 5.92 | 11.3 | 50.0 | 38.5 | 26.3 | 520 |
| E-1 | 1 | 3.72 | 4.59 | 9.22 | 12.7 | 5.99 | 149 |
| E-1 | 3 | 1.57 | 6.24 | 5.79 | 11.4 | 5.49 | 97 |
| E-1 | 12 | 4.25 | 7.48 | 13.0 | 13.1 | 23.7 | 291 |
| E-1 | 24 | 5.22 | 11.7 | 32.5 | 33.2 | 16.9 | 362 |
| E-2 | 1 | 3.33 | 5.40 | 12.7 | 15.3 | 11.7 | 205 |
| E-2 | 3 | 1.64 | 3.00 | 6.20 | 8.22 | 9.36 | 127 |
| E-2 | 12 | 1.71 | 3.50 | 10.7 | 18.3 | 10.1 | 160 |
| E-2 | 24 | 6.90 | 16.4 | 29.5 | 40.2 | 9.03 | 298 |
| E-3 | 1 | 1.63 | 1.91 | 5.92 | 11.8 | 12.4 | 140 |
| E-3 | 3 | 1.29 | 2.24 | 4.93 | 13.4 | 5.30 | 85 |
| E-3 | 12 | 4.50 | 6.62 | 17.9 | 33.5 | 2.73 | 176 |
| E-3 | 24 | 3.73 | 5.36 | 18.1 | 13.1 | 21.0 | 311 |
