# Supplementary material for: Comparative Analysis of Fecal Microbiota in Infants with and without Eczema
Source: PLoS One. 2010 Apr 1;5(4):e9964. doi: 10.1371/journal.pone.0009964 (PMC2848600; doi:10.1371/journal.pone.0009964)
Supplement: Table S2 — List of HOPE primers used in this study. (0.05 MB DOC) [file pone.0009964.s003.doc]

**Table S2.** HOPE primers targeting *Bifidobacterium*, *Enterococcus* and Enterobacteriaceae at different taxonomical levels.

| **Tube no.** | **Primer name** | **Targeted bacteria** | **Primer sequence (5’-3’)** | **Extended ddNTP** | **PolyA tail**  **(nt)** | **Total length (nt)** |
| --- | --- | --- | --- | --- | --- | --- |
| 1 | Eub338Ia | Domain Bacteria | GCTGCCTCCCGTAGGAG | T | 23 | 40 |
|  | Bif660 | Genus *Bifidobacterium* | CCACCGTTACACCGGGAATTCCAG | T | 10 | 34 |
|  | Bifgp1250 | *B. dentium* and *B. bifidum* group | GTCGCCATGTCGCATCCCGC | T | 8 | 28 |
|  | Bifgp272 | *B. angulatum* and *B. catenulatum* group | GCCGGCTACCCGTCGTAGGCTC | G | 0 | 22 |
|  | Bifgp442 | *B. adolescentis* and *B. ruminantium* group | CCGAAGGGCTTGCTCCCAG | T | 0 | 19 |
|  | Blon1004 | *B. longum* group | AGCCGTATCTCTACGACCGT | C | 0 | 20 |
|  |  |  |  |  |  |  |
| 2 | Eub338Ia | Domain Bacteria | GCTGCCTCCCGTAGGAG | T | 23 | 40 |
|  | Ent834 | Family Enterobacteriaceae | GAAGCCACGCCTCAAGGGCACAA | C | 0 | 23 |
|  | Enc131 | Genus *Enterococcus* | CCCCTTCTGATGGGCAGG | T | 0 | 18 |
|  | Esc473 | *Escherichia* and *Shigella* group | CGGGTAACGTCAATGAG | C | 3 | 20 |
|  |  |  |  |  |  |  |
