# Supplementary material for: Comparative Analysis of Fecal Microbiota in Infants with and without Eczema
Source: PLoS One. 2010 Apr 1;5(4):e9964. doi: 10.1371/journal.pone.0009964 (PMC2848600; doi:10.1371/journal.pone.0009964)
Supplement: Figure S1 — Rarefraction curves. (1.31 MB PDF) [file pone.0009964.s004.pdf]

Figure S1A

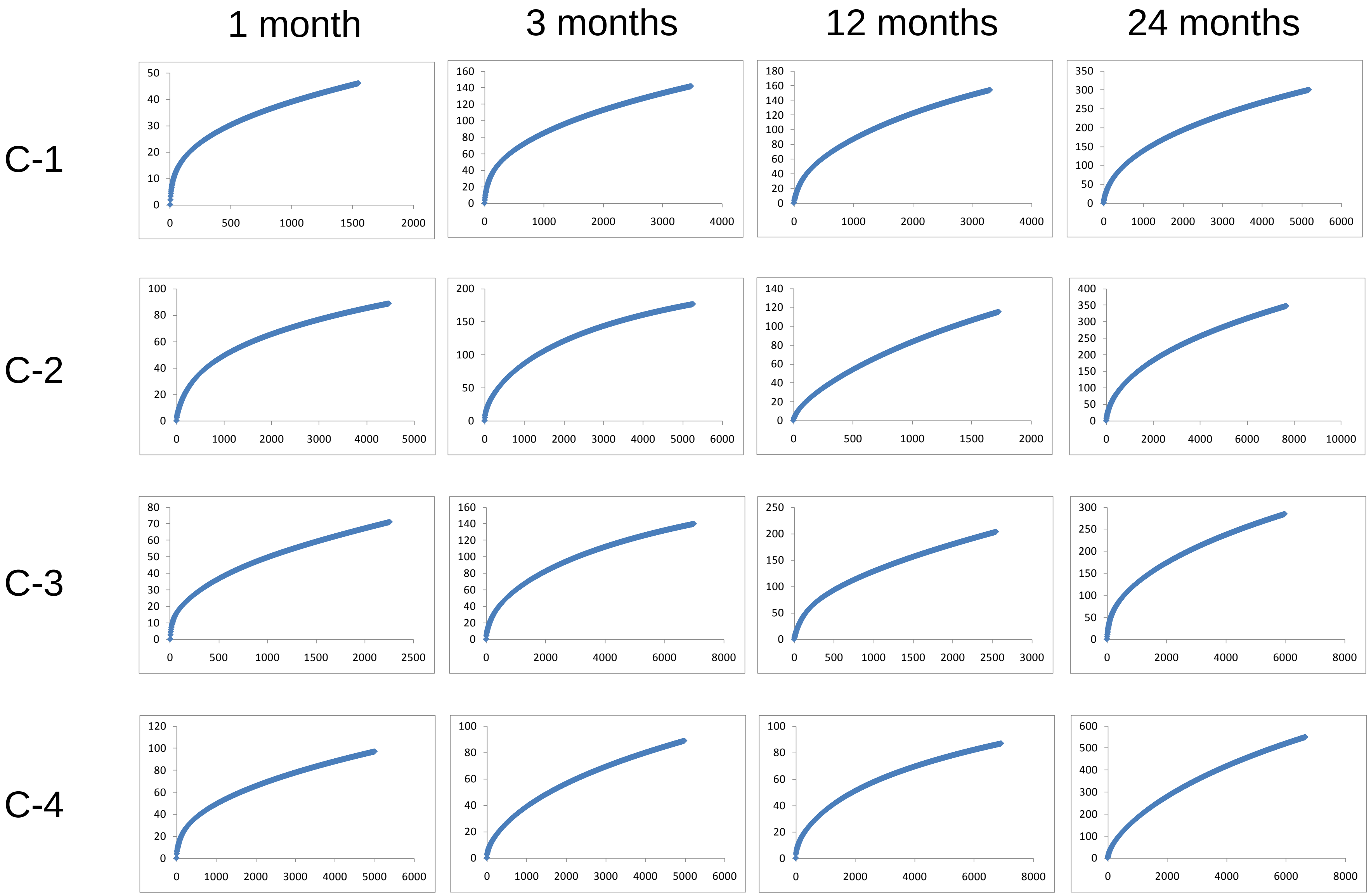
1 month
3 months
12 months
24 months
C-1
C-2
C-3
C-4
0 10 20 30 40 50
0 500 1000 1500 2000
0 20 40 60 80 100 120 140 160
0 1000 2000 3000 4000
0 20 40 60 80 100 120 140 160 180
0 1000 2000 3000 4000
0 50 100 150 200 250 300 350
0 1000 2000 3000 4000 5000 6000
0 20 40 60 80 100
0 1000 2000 3000 4000 5000
0 50 100 150 200
0 1000 2000 3000 4000 5000 6000
0 20 40 60 80 100 120 140
0 500 1000 1500 2000
0 50 100 150 200 250 300 350 400
0 2000 4000 6000 8000 10000
0 10 20 30 40 50 60 70 80
0 500 1000 1500 2000 2500
0 20 40 60 80 100 120 140 160
0 2000 4000 6000 8000
0 50 100 150 200 250
0 500 1000 1500 2000 2500 3000
0 50 100 150 200 250 300
0 2000 4000 6000 8000
0 20 40 60 80 100 120
0 1000 2000 3000 4000 5000 6000
0 20 40 60 80 100
0 1000 2000 3000 4000 5000 6000
0 20 40 60 80 100
0 2000 4000 6000 8000
0 100 200 300 400 500 600
0 2000 4000 6000 8000

Figure S1B

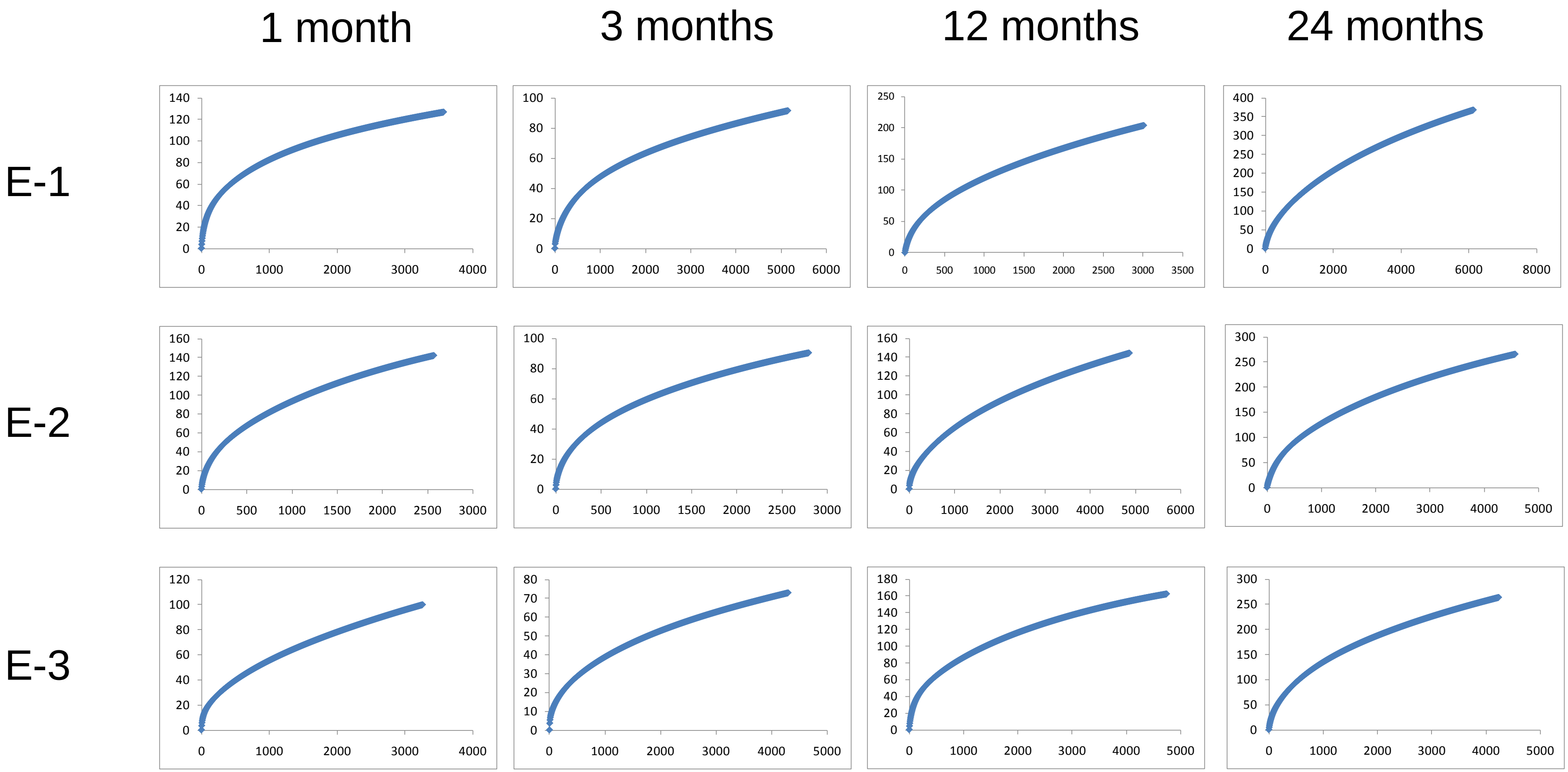

Figure S1. Rarefraction curves. The number of operational taxonomic units (OTUs) defined at 97% similarity was denoted along vertical y-axis, and plotted against the number of 16S pyrotags sequenced (x-axis). (A) Rarefraction curves for non-eczema control infants C-1 to C4. (B) Rarefraction curves for eczema infants, E-1 to E-3.
